# DHFR silence alleviated the development of liver fibrosis by affecting the crosstalk between hepatic stellate cells and macrophages

**DOI:** 10.1111/jcmm.16935

**Published:** 2021-10-09

**Authors:** Yu Peng, Zedong Li, Sheng Chen, Jun Zhou

**Affiliations:** ^1^ Department of General Surgery The Second Xiangya Hospital Central South University Changsha China; ^2^ Department of Emergency Medicine The Second Xiangya Hospital Central South University Changsha China

**Keywords:** exosomes, hepatic stellate cells, liver fibrosis, macrophages

## Abstract

Liver fibrogenesis is a dynamic cellular and tissue process which has the potential to progress into cirrhosis of even liver cancer and liver failure. The activation of hepatic stellate cells (HSCs) is the central event underlying liver fibrosis. Besides, hepatic macrophages have been proposed as potential targets in combatting fibrosis. As for the relationship between HSCs and hepatic macrophages in liver fibrosis, it is generally considered that macrophages promoted liver fibrosis via activating HSCs. However, whether activated HSCs could in turn affect macrophage polarization has rarely been studied. In this study, mRNAs with significant differences were explored using exosomal RNA‐sequencing of activated Lx‐2 cells and normal RNA‐sequencing of DHFR loss‐of‐function Lx‐2 cell models. Cell functional experiments in both Lx‐2 cells and macrophages animal model experiments were performed. The results basically confirmed exosomes secreted from activated HSCs could promote M1 polarization of macrophages further. Exosome harbouring DHFR played an important role in this process. DHFR silence in HSCs could decrease Lx‐2 activation and M1 polarization of M0 macrophages and then alleviate the development of liver fibrosis both in vitro and vivo. Our work brought a new insight that exosomal DHFR derived from HSCs had a crucial role in crosstalk between HSCs activation and macrophage polarization, which may be a potential therapeutic target in liver fibrosis.

## 1 INTRODUCTION

Liver fibrosis would gradually develop into liver cancer and liver failure which was caused by various factors.^1^, ^2^ It is characterized by excessive deposition of extracellular matrix (ECM) proteins, the major source of which are activated hepatic stellate cells (HSCs).^3^ In the normal liver, HSCs are quiescent.^4^ Once liver injury occurs, HSCs are activated. HSC activation results from the inflammatory activity of liver immune cells, predominantly macrophages.^5^ Activated HSCs (aHSCs) can secrete chemokines and chemokine receptors to recruit macrophages into injured liver to initiate innate immune response, and recruited macrophages promote fibrosis by releasing cytokines and chemokine that activate HSCs and exacerbate inflammation.^6^ There is a positive feedback loop between inflammatory and fibrogenic cells, which in turn results in more severe fibrosis.^1^

Macrophages are dynamic and highly plastic cells which played important roles in liver inflammatory response: uncommitted macrophages (M0) depending on local microenvironment polarize into classically activated macrophages (M1) or alternatively activated macrophages (M2) with specific functions.^7^ M1 macrophages mediate tissue damage and initiate inflammatory responses, while M2 macrophage is an essential player in the resolution of inflammation, so M1/M2 macrophage balance polarization governs the fate of an organ in inflammation or injury.^8^ Exosomes harbour various biomolecules, including lipid, proteins, DNAs, mRNAs and non‐coding RNAs, which can be transferred to recipient cells and modulate their functions^9^, ^10^ Exosomes are known as important mediators of cell‐cell communication.^11^ In liver, various types of cell can either release or receive exosomes in cell‐cell communication, including HSCs and macrophage. Exosomes involve in various aspects of liver physiology and pathology and in the progress of liver diseases, like HSCs activation and participating in liver fibrosis.^12^ Exosomes derived from qHSCs or aHSCs possess anti‐fibrotic and pro‐fibrotic properties respectively.^13^ Exosomes derived from natural killer cells can inhibit HSCs activation and liver fibrosis.^14^, ^15^ Macrophage also could take up exosomes from other cells which leading to macrophages activation,^16^ polarization which directly influence macrophage function.^17^, ^18^, ^19^, ^20^, ^21^, ^22^ However, whether aHSCs‐derived exosomes could affect macrophage polarization and further regulate the progression of liver fibrosis have not been fully studied.

Our research found aHSCs‐derived exosomes promoted M1 macrophage polarization and inhibited M2a polarization. Then, exosome‐RNA‐sequencing (Exo‐RNA‐seq) was conducted and bioinformatic analysis results showed that dihydrofolate reductase (DHFR) was one of the most significant up‐regulated genes in exosomes derived from activated Lx‐2 cells when compared with quiescent Lx‐2 cells which suggested it could participate in liver fibrosis.^23^ DHFR is a crucial target in anticancer drug development which plays key roles in the nucleic acid biosynthesis. Through TCGA online database retrieval, DHFR was found to be significantly up‐regulated in LIHC primary tissues and it was negatively correlated with the overall survival rate of LIHC patients. However, there is no relevant research on whether DHFR is involved in the regulation of fibrosis. Thus, DHFR‐silenced Lx‐2 cell model was established to explore the biological function of DHFR in Lx‐2 activation and interaction with macrophages. DHFR silence in Lx‐2 cells not only reduced the DHFR in exosomes, but also decreased the activation and proliferation ability of TGF‐β‐induced Lx‐2 cells. On the other hand, when M0 macrophages absorbed exosomes secreted by DHFR‐silenced Lx‐2, M1 macrophage polarization was decreased. In mice liver fibrosis model induced by CCl‐4, collagen fibre decreased obviously in DHFR knock‐down mice. It demonstrated that DHFR functioned as an fibrogenic gene in vitro and in vivo in liver fibrosis. Subsequently, RNA‐sequence (RNA‐seq) was performed again in the DHFR‐silenced Lx‐2 cells in which INPP5D was found to be one of a key downstream target gene of DHFR.

In brief, our results brought a new insight that aHSCs‐derived exosomal DHFR has a crucial role in crosstalk between HSC activation and macrophage polarization, which may be a potential therapeutic target in liver fibrosis.

## 2 MATERIALS AND METHODS

### 2.1 Ethics statement

Animal and human studies were approved by the ethics committee of Xiangya Second Hospital of Central South University (Project number: 2020161; 2020R113). All selected patients were treated based on the Liver Disease Treatment Guide.^24^ Additionally, the patients' consent and the approval were all obtained (Zhou et al., 2019). During the process of study, the ethical standards of the Committee on Human Experimentation and the Helsinki Declaration were strictly obeyed. Liver tissues from patients with liver fibrosis and cirrhosis were diagnosed by liver biopsy. Both tumour tissues and bile samples were collected from surgical operations.

Animal care in the research was conducted according to the Guide for the Care and Use of Laboratory Animals enacted by the US National Institutes of Health and was approved by the Animal Research Committee of Center of Central South University.

### 2.2 Lx‐2 cell activation and macrophage polarization

Lx‐2 cells were cultured in DMEM medium containing 2% foetal bovine serum for 24 h. After starvation for 12 h, Lx‐2 cells were treated with 5 ng/ml TGF‐β for another 48 h.

Peripheral blood mononuclear cells (PBMC) were isolated from healthy volunteers using a human PBMC isolation Kit (Solarbio) and cultured in DMEM supplemented with 10% FBS. Non‐adherent cells were removed after 3 h,^19^ and the monocytes (adherent cells) were cultured in DMEM supplemented with macrophage colony‐stimulating factor (M‐CSF, 50 ng/ml, PeproTech) for 6 days. IFN‐γ (50 ng/ml, Peprotech) and IL‐4 (50 ng/ml, Peprotech) were used for M0 to M1 and M2 polarization, respectively. Then, human M0, M1 and M2 macrophages were subjected to co‐culture system.

### 2.3 Exosome isolation and identification

Lx‐2 cells were cultured in DMEM medium containing 10% FBS (exosome‐depleted). Lx‐2‐exosomes were extracted using SBI Exosome Isolation Reagent (ExoQuick‐TC, Cat.no: EXOTC50A‐1) according to the manufacturer's instructions. Exosomal markers, CD81 and TSG101 were used to verify exosomes with Western blot. The exosomes were photographed by electron microscope (Hitachi, HT7700).

### 2.4 Exosome uptake labelling

To confirm exosomes were absorbed by receptor macrophages, PKH67 mixer (Cat.no CGLDIL, Sigma, in Diluent C) was used to label exosomes from 1.5 × 10^6^ cells according to the manufacturer's instructions. The stained cells were observed and photographed with a fluorescence confocal microscope (Zeiss LSM880).

### 2.5 RNA‐sequencing

As to Exo‐RNA‐seq, total RNAs were extracted from the exosomes of Lx‐2 culture supernatant. Besides, RNAs were extracted from exosomes from activated (Lx‐2‐TGF‐β) and quiescent Lx‐2 cells (Lx‐2‐C), and then the RNA‐seq and relative bioinformatics analysis were performed in Kaitai Biotech and Science & Well biotech. The differentially expressed genes (DEGs) between exosomes derived from Lx‐2‐TGF‐β and Lx‐2‐C groups were selected by the difference multiples (|log2FoldChange| > 1) and significance (*p* < 0.05). The subsequent RNA‐seq in DHFR‐silenced Lx‐2 cells was also implemented according to the above methods.

### 2.6 qRT‐PCR analysis

To detect mRNA relative expression, total RNAs were extracted from cells and tissue samples using RNAsimple Total RNA Kit (Tiangen); then, complementary DNA was synthesized using RevertAid First Strand cDNA Synthesis Kit(K1622) (ThermoFisher) and reverse transcription was followed by RT‐PCR using the SuperReal PreMix Plus (SYBR Green) (Tiangen). Primers used in this study are shown in Table 1.

**TABLE 1:** Primer sequence information used in this study

Gene	Primer‐Forward	Primer‐Reverse
α‐SMA	ACCCTTCAGCTTTCAGCTTCC	CACCATCACCCCCTGATGTCTG
Collagen‐I‐α1	GGACACAGAGGTTTCAGTGG	CAGTAGCACCATCATTTCCACG
CTGF	CTGGTCCAGACCACAGAGTG	TGCCCTTCTTAATGTTCTCTTCCA
IL‐1β	ATGATGGCTTATTACAGTGGCAA	GTCGGAGATTCGTAGCTGGA
CCR7	TGAGGTCACGGACGATTACAT	GTAGGCCCACGAAACAAATGAT
MRC1	TCCCTCAGAAAGTGATGTGCC	AGCCGATCCACAATTCGTCA
TIMP3	ACCGAGGCTTCACCAAGATG	CATCATAGACGCGACCTGTCA
TNF‐α	GTTGTAGCAAACCCTCAAGCTG	GAGGTACAGGCCCTCTGATG
IL‐6	ACTCACCTCTTCAGAACGAATTG	CCATCTTTGGAAGGTTCAGGTTG
DHFR	AACTCAAGGAACCTCCACAAG	ACTGCCACCAACTATCCAGAC
Timp‐1	CCAGAACCGCAGTGAAGAGT	TCTGGTAGTCCTCAGAGCCC
Col3α1	CCTTCTACACCTGCTCCT	CTTCCTGACTCTCCATCCT
Col4α1	AACAACGTCTGCAACTTCGC	CTTCACAAACCGCACACCTG
INPP5D	CCAAGAAGATCACGTCCTGGT	CGTGTGGATGGCGACTGTT
β‐actin	TTCCTTCCTGGGCATGGAGTC	TCTTCATTGTGCTGGGTGCC

### 2.7 Western blot assay

For the sake of detecting the expression of fibroblast activation markers and inflammatory markers of macrophages, Western blot was carried out as previously described.^25^ Amount of the protein of interest was normalized to the densitometric units of β‐actin. Antibodies used were shown in Supplementary Material.

### 2.8 Cell survival assay

In order to evaluate the viability of Lx‐2 cells after DHFR silence, the Cell Counting Kit‐8 (CCK‐8) assay was used to assess the cell survival ability until 96 h as previously described (Cat.no K1018; APExBio).^26^ To evaluate the viability of M0 macrophages absorbed different exosomes, CCK‐8 was performed again at 24 and 48 h respectively.

### 2.9 Immunofluorescence of M0 macrophages

M0 macrophages were incubated with primary antibodies for IL‐12 (1:100, abcam, ab133725), IRF5 (dilution 1:1000, abcam, ab181553) overnight at 4℃ before washing with PBS. Fluorescein‐labelled secondary antibody was used at RT in the dark for 2 h (dilution 1:500). Slides were mounted in mounting media with DAPI for 1 h at RT. After washing with PBS, the slides were covered with DABCO and images were captured by fluorescence microscopy (AE31; Motic).

### 2.10 H&E staining and Masson Trichrome staining

6–8‐week aged male C57BL/6 mice were purchased from the Promab biotech Co. Ltd. Mice were randomly divided into 3 groups (*n* = 6), and all mice were maintained under standard conditions and diet. Experimental hepatic fibrosis was induced by CCl‐4 (10% in olive oil, 2 ml/kg, twice a week for 6 weeks). Paraformaldehyde‐fixed liver tissue sections were stained with haematoxylin and eosin (H&E) and Masson's Trichrome staining to evaluate liver fibrosis. Collagen deposition was quantitatively analysed using the following formula: collagen area/total area × 100%.

### 2.11 Statistical analysis

All tests were set up in triplicate. Statistical analysis was performed and presented with Graphpad Prism 7.0 software (GraphPad Software). Differences between two independent groups were evaluated by Student's *t* tests. Differences for multiple comparisons were calculated using one‐way ANOVA. *p* < 0.05 was considered significant differences. Data are presented as mean ± SD.

## 3 RESULTS

### 3.1 Exosomes derived from activated Lx‐2 induced M1 macrophage polarization of M0 macrophage

**FIGURE 1:**
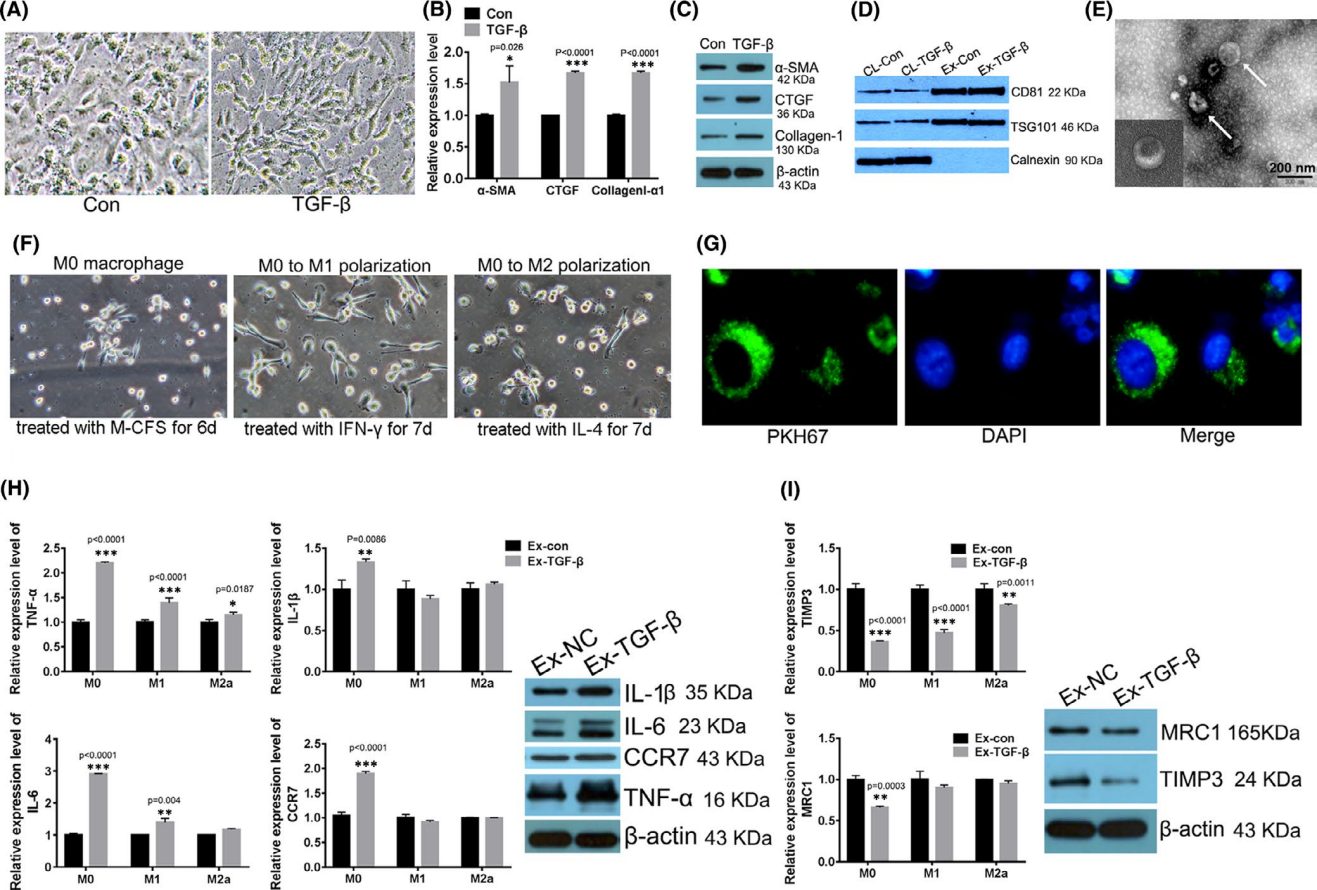
Exosomes derived from activated Lx‐2 induced M1 macrophage polarization of M0 macrophage. (A) The typical pictures of cell morphology of Lx‐2 treated with TGF‐β, the magnification was 200 times. The expression of Lx‐2 activation markers was evaluated using qPCR (B) and Western blot (C). *p < 0.05, ***p < 0.0001 vs. Con group. (D) The exosome protein markers were detected using Western blot. (E) Representative TEM image of exosomes isolated from Lx‐2 cells, white arrows indicated exosomes, the scale bar was 200 nm, the small field in the lower left corner showed the morphology of a single exosome taken in addition, which was independent of the exosomes indicated by the white arrows. (F) The monocytes were treated with 50 ng/ml M‐CSF for 6 days to generate M0 macrophages; then, 50 ng/ml IFN‐γ and 50 ng/ml IL‐4 were used for M0 to M1 and M2 polarization, respectively. The typical pictures of cell morphology of different macrophages were taken, and the magnification was 200 times. (G) PKH67 staining to verify exosome uptake using scanning electron microscope. (H) The expression of M1 macrophage markers was evaluated using QPCR and Western blot. *p < 0.05, **p < 0.01, ***p < 0.0001 vs. Ex‐con group. (I) The expression of M2a macrophage markers was evaluated using QPCR and Western blot, **p < 0.01, ***p < 0.0001 vs. Ex‐con group

To evaluate the effect of exosomes derived from aHSCs on macrophage polarization, TGF‐β‐treated Lx‐2 was used as exosome donor cell model in vitro. After 48 h of TGF‐β (5 ng/ml) treatment, the cell morphology changed from irregular to long spindle (Figure 1A). Then, HSC activation markers were detected by QPCR and Western blot. The results showed that α‐SMA, collagen‐I and CTGF were all enhanced significantly in TGF‐β‐treated group when compared with control group in both mRNA levels and protein levels (Figure 1B,C). Then, exosomes secreted by activated Lx‐2 were extracted and identified after the Lx‐2 was confirmed to be activated from morphology and molecular markers checking. Exosome markers were detected in cell lysates and exosomes. The result showed that the content of exosome marker proteins CD81 and TSG101 in exosomes was significantly higher than those in cell lysates. Calnexin, as a negative control, had no significant expression in exosome samples (Figure 1D). Exosome morphology was examined using TEM which were showed membrane‐bounded, spherical shape vesicles with a size range of 50–100 nm (Figure 1E) and was consistent with the typical morphology of exosomes reported previously.

In order to investigate the effect of exosomes secreted by activated Lx‐2 on macrophage transpolarization after being absorbed and internalized by macrophages, firstly, PBMC cells from healthy volunteers were induced into M0 macrophage; then, were polarized to M1 and M2a macrophagy. The morphology of 3 types of macrophages was confirmed under light microscopy (Figure 1F). Then, the exosomes secreted by activated Lx‐2 were co‐cultured with three different macrophages. The result showed that after being absorbed by macrophages, exosomes labelled by PKH67 (green fluorescence) surround the nuclear region (Figure 1G). Subsequently, the expression of macrophage subtype markers was detected by QPCR. The results demonstrated that when exosomes derived from activated Lx‐2 were uptook by M0, M1 and M2a macrophage, the M1 markers (CCR7, IL‐β, IL‐6 and TNF‐α) were all significantly up‐regulated (Figure 1H); on the contrary, the M2a marker (MRC1 and TIMP3) decreased significantly in M0 macrophagy (Figure 1I). In addition, the expression of these markers in M1 and M2a macrophages was irregular and inconsistent. Therefore, Western blot was used to detect the markers of M1 and M2a subtypes in M0 macrophages absorbed exosomes secreted by Lx‐2. At the protein level, M1 subtype markers CCR7, IL‐β, IL‐6 and TNF‐α were all enhanced (Figure 1J) while the markers of M2a (MRC1 and TIMP3) decreased significantly (Figure 1K) in M0 macrophage‐absorbed exosomes derived from activated Lx‐2 (Ex‐TGF‐β) when compared with which absorbed exosomes derived from inactivated Lx‐2 (Ex‐NC). Based on the above results, it was speculated that exosomes derived from activated Lx‐2 induced M1 macrophage polarization of M0 macrophage.

### 3.2 DHFR was enriched in exosomes derived from activated Lx‐2 cells

**FIGURE 2:**
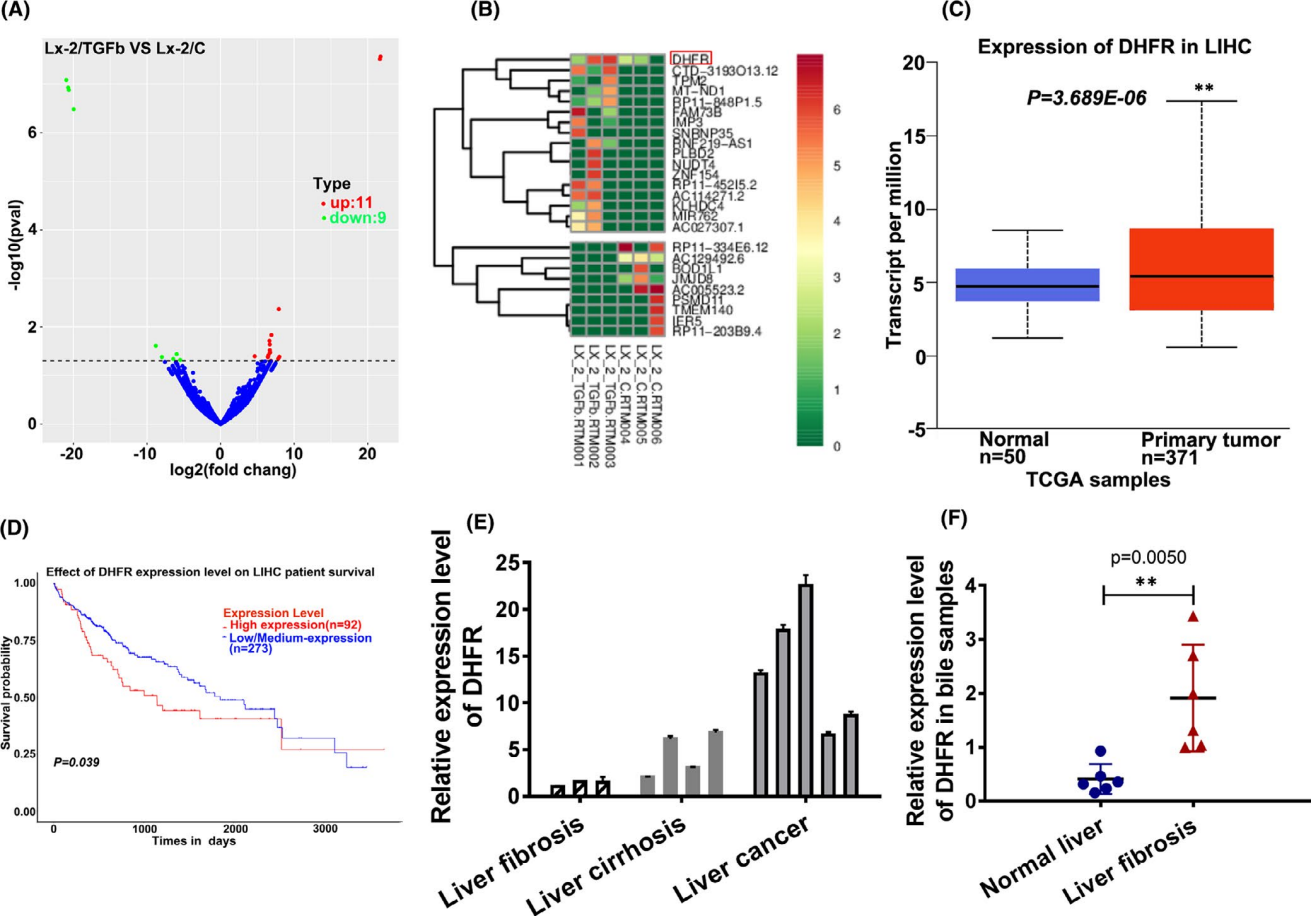
DHFR was enriched in exosomes derived from activated Lx‐2 cells. (A) Volcano plot of differential expression genes from exosomal RNA‐Seq in exosomes derived from activated Lx‐2 cells (Lx‐2‐TGFb) vs. quiescent Lx‐2 cells (Lx‐2C). (B) Cluster by groups to show significantly deregulated exosomal mRNAs upon exosomes derived from activated Lx‐2 cells (Lx‐2‐TGFb) vs. quiescent Lx‐2 cells (Lx‐2C), (absolute value of Log2fc > 1.0 and *p* < 0.05). (C) Expression of DHFR in LIHC samples from TCGA database. **p < 0.01 vs. normal tissue group. (D) Effect of DHFR expression on LIHC patient survival probability from TCGA data. (E) The relative mRNA level of DHFR in liver fibrosis, liver cirrhosis and liver cancer tissue samples collected clinically. (F) The relative mRNA level of DHFR in bile samples collected during operation, **p < 0.01 vs. normal group

Exo‐RNA‐seq was performed on exosomes derived from activated Lx‐2 cells and quiescent Lx‐2 cells. Although effective information captured was not rich, 11 significant up‐regulated genes and 9 down‐regulated genes were obtained (*p* < 0.05, Absolute value of Log Fc > 1.0) (Figure 2A). Through cluster analysis (Figure 2B), DHFR was found to be one of the most significant up‐regulated genes in exosomes derived from activated Lx‐2 cells (Lx‐2‐TGFb) vs. quiescent Lx‐2 cells (Lx‐2C). Because there is a progressive correlation from liver fibrosis, liver cirrhosis to liver cancer, DHFR attracted our attention. Through TCGA online database retrieval (http://ualcan.path.uab.edu/analysis.html), DHFR was found to be significantly up‐regulated in LIHC primary tissues (*n* = 371) than in normal liver tissues (*n* = 50). Furthermore, by analysing the overall survival rate of LIHC patients (*n* = 365) with differential expression of DHFR, it was found that the DHFR expression was negatively correlated with the overall survival rate of LIHC patients (*p* = 0.039) (Figure 2C,D). These results suggested that DHFR may play an important role in the process of LIHC. Therefore, 3 cases of liver fibrosis tissues, 4 cases of liver cirrhosis tissues and 5 cases of liver cancer tissues were collected from minimally invasive surgery and general surgery of the Xiangya second hospital of central south university, and DHFR expression was detected using qPCR detection. The results showed that the expression level of DHFR increased gradually from liver fibrosis to liver cirrhosis and then to liver cancer at least in the limited collected samples (Figure 2E). Subsequently, DHFR expression was verified again; in collected bile samples, it was found that DHFR enhanced obviously in bile samples of liver fibrosis patients when compared with bile samples from normal volunteers (Figure 2F).

### 3.3 DHFR silence in Lx‐2 alleviated activation of HSCs and M1 macrophage polarization of M0 macrophage

**FIGURE 3:**
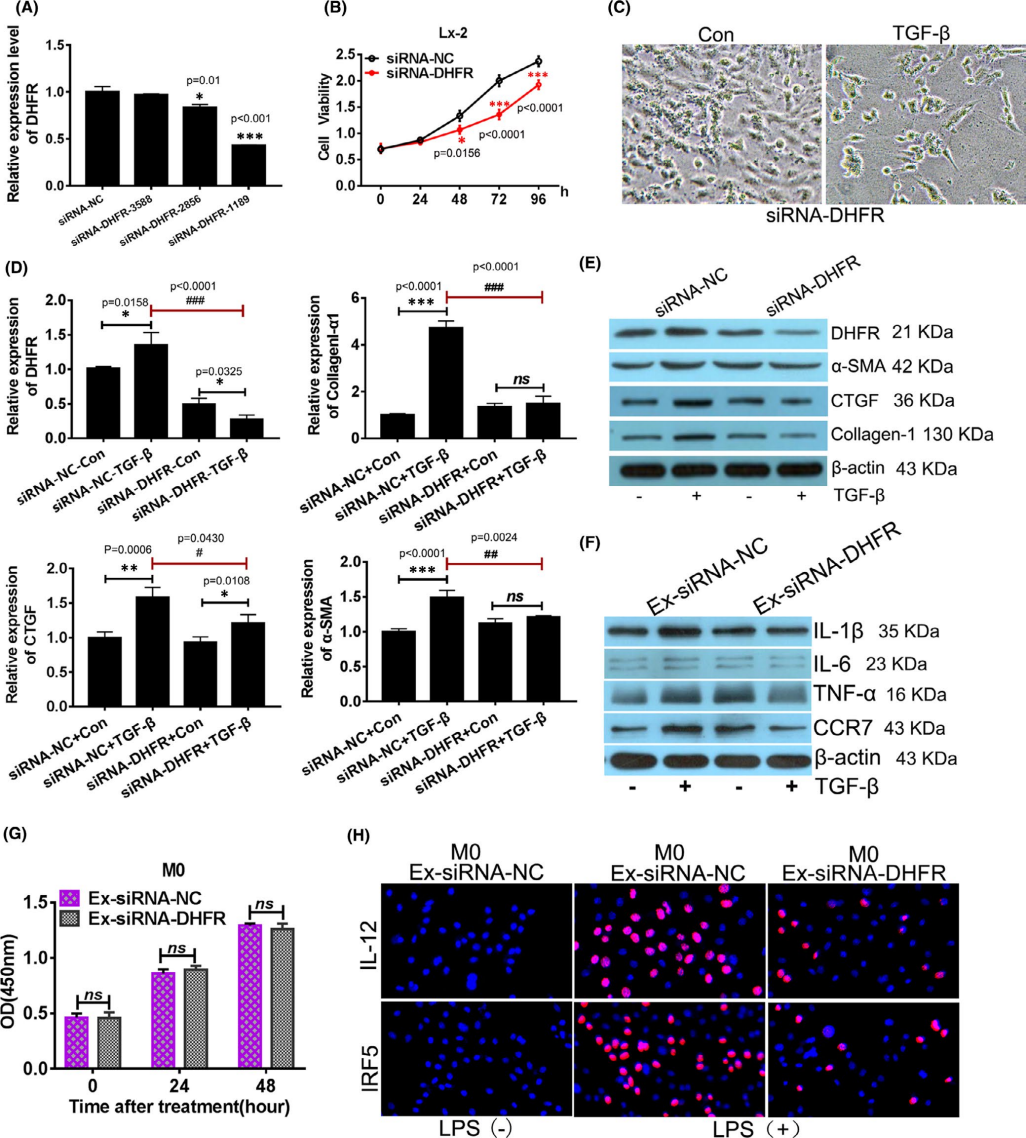
DHFR silence in Lx‐2 alleviated activation of HSCs and M1 macrophage polarization of M0 macrophage. (A) Selection and validation of DHFR effective siRNA sequences using QPCR, *p < 0.05, ***p < 0.001 vs. siRNA‐NC group. (B) Cell viability of DHFR‐silenced LX‐2 cells, *p < 0.05, ***p < 0.0001 vs. siRNA‐NC cells. (C) The typical pictures of cell morphology of DHFR‐silenced Lx‐2 treated with TGF‐β, the magnification was 200 times. The relative mRNA (D) and protein expression (E) of DHFR and Lx‐2 activation markers were tested in DHFR‐silenced Lx‐2 cells treated with TGF‐β. *^, #^ *p* < 0.05, **^,##^ *p* < 0.01, ***^, ###^ *p* < 0.0001 between the two appointed groups. (F) The protein expression of M1 macrophage markers was evaluated using Western blot in DHFR‐silenced Lx‐2 cells treated with TGF‐β. (G) Cell viability of M0 macrophage‐absorbed exosomes from DHFR‐silenced LX‐2 cells. (H) M1 polarization was detected by immunofluorescence of M0 macrophage‐absorbed exosomes from DHFR‐silenced LX‐2 cells, the magnification was 200 times

To investigate whether DHFR plays a role in the regulation of Lx‐2 activation, DHFR was knocked‐down in Lx‐2 by siRNA interference. Three different siRNA sequences were designed and synthesized and transiently transfected into Lx‐2. QPCR was used to verify the loss‐of‐function Lx‐2 cell model of DHFR. It was demonstrated that only siRNA‐1189 achieved effective interference which was selected for subsequent experiments (Figure 3A). Then, the survival ability of activated Lx‐2 was evaluated and it was found that when DHFR was silenced in Lx‐2, the cell viability reduced significantly from 48 h after TGF‐β treatment when compared with siRNA‐NC group cells (Figure 3B). At the same time, we also observed that not only the cell number decreased, but also the morphological changes of activated Lx‐2 were no longer obvious under the microscope (Figure 3C). Markers of hepatic stellate cell activation were detected in different Lx‐2 cell models using QPCR and Western blot (Figure 3D,E). When parental Lx‐2 cells (siRNA‐NC‐Con) were treated with TGF‐β, α‐SMA, collagen‐I and CTGF were all up‐regulated significantly when compared with the parental Lx‐2 cells without TGF‐β treatment. But in DHFR loss‐of‐function Lx‐2 cells (siRNA‐DHFR‐Con), the expression alteration of α‐SMA, collagen‐I and CTGF was no longer significant even with a certain degree of decline. In particular, there was significant down‐regulation of α‐SMA, collagen‐I and CTGF expression in DHFR loss‐of‐function Lx‐2 cells (siRNA‐DHFR‐Con) when compared with parental Lx‐2 cells (siRNA‐NC‐Con) with TGF‐β treatment. The results suggested that DHFR silence could alleviate activation of Lx‐2.

What effect did it have on the transpolarization of M0 macrophages absorbed the exosomes secreted by Lx‐2 cells with DHFR silence? Exosomes derived from the parental Lx‐2 cells (Ex‐siRNA‐NC) and Lx‐2 cells with DHFR silence (Ex‐siRNA‐DHFR) were absorbed by M0 macrophages respectively. Then, the effect of different exosomes on the cell viability of M0 macrophages was determined by the CCK‐8 assay. Notably, it was observed that different exosome uptake did not affect.

M0 macrophage viability in vitro (Figure 3G). Meanwhile, our immunostaining experiment with anti‐IL‐12/anti‐IRF5 and Western blot detection of other M1 macrophage markers suggested that when M0 macrophage uptook exosomes secreted from Lx‐2 cells with DHFR silence (Ex‐siRNA‐DHFR), the M1 macrophages polarization was obviously suppressed when compared with the parental Lx‐2 cells (Ex‐siRNA‐NC) group (Figure 3F,H). The results suggested that DHFR silence in Lx‐2 could inhibit M1 macrophage polarization of M0 macrophage.

### 3.4 DHFR knock‐down alleviated CCL‐4‐induced hepatic fibrosis in mice

**FIGURE 4:**
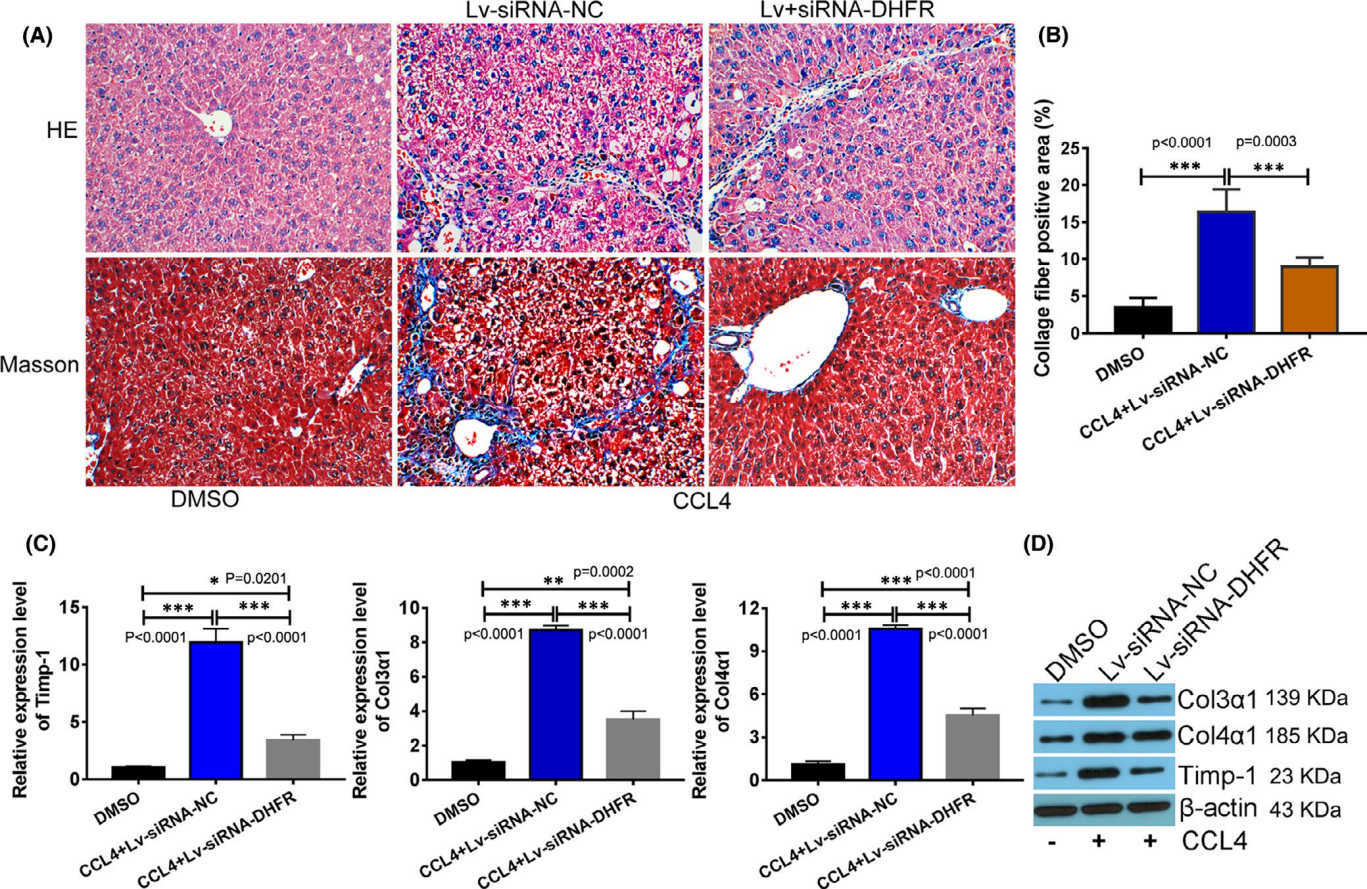
DHFR knock‐down alleviated CCL‐4‐induced hepatic fibrosis in mice. Mouse liver samples dyed with HE or Masson's trichrome (A) and semi‐quantitative measurement of Masson's staining, the magnification was 200×. (B), ***p < 0.001 between the indicated groups. Effects of CCL‐4 and DHFR silence on hepatic protein expression of TIMP‐1 and collagen 3α and 4α using (C) QPCR and (D) Western blotting, respectively. *p < 0.05, **p < 0.01, ***p < 0.0001 between indicated groups

Due to the obvious advantages over bile duct ligation (BDL) method reported previously, CCL‐4 was selected to induce the experimental mice model in the further mechanism research. In our study, the DHFR‐silenced lentivirus (Lv‐siRNA‐DHFR) was packaged and injected into C57BL/6 mice, and the efficacy of DHFR knock‐down was tested at week 2. HE staining showed that the fibrosis phenotype of liver tissues of the group injected with DHFR‐silenced lentivirus (Lv‐siRNA‐DHFR) was significantly alleviated. The Masson trichrome results also presented that the degree of collagen deposition in Lv‐siRNA‐DHFR group was decreased obviously (Figure 4A). Collagen fibre‐positive area was calculated and analysed with IPP6.0 software. The results showed that after CCL‐4 modelling, the collagen fibre‐positive area in liver tissues of Lv‐siRNA‐NC group was significantly increased, while that of the group injected with DHFR‐silenced lentivirus (Lv‐siRNA‐DHFR) was significantly decreased when compared with Lv‐siRNA‐NC group (Figure 4B). Correspondingly, collagen‐related markers were detected by QPCR and Western blot. It was also showed that the expression of Timp‐1, Col3α1 and Col4α1 was all significantly increased in CCL‐4 group with siRNA‐NC lentivirus (Lv‐siRNA‐NC), while that of the group injected with DHFR‐silenced lentivirus (Lv‐siRNA‐DHFR) was significantly down‐regulated when compared with Lv‐siRNA‐NC group (Figure 4C,D). These results suggested that DHFR knock‐down alleviated CCL‐4‐induced hepatic fibrosis in mice to some extent.

### 3.5 INPP5D was one of the downstream negative regulators of DHFR in Lx‐2 cells

**FIGURE 5:**
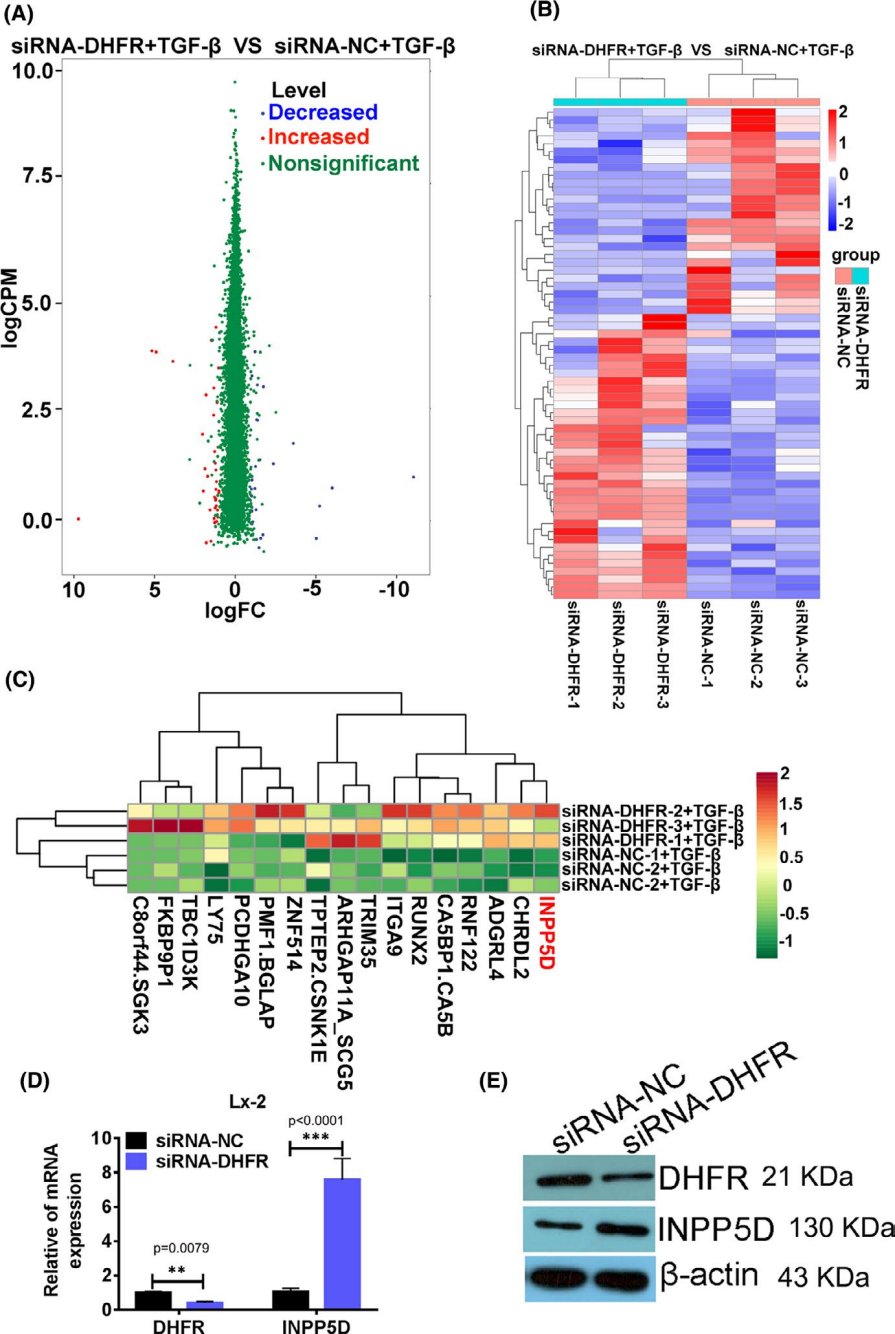
INPP5D was one of the downstream negative regulators of DHFR in Lx‐2 cells. (A) Volcano plot of differential expression genes from RNA‐Seq in activated Lx‐2 cells with (siRNA‐DHFR) or without (siRNA‐NC) DHFR knock‐down. (B) Cluster by samples to show significantly deregulated mRNAs between activated Lx‐2 cells with (siRNA‐DHFR) or without (siRNA‐NC) DHFR knock‐down, (absolute value of Log2fc > 1.0 and *p* < 0.05). (C) Cluster by samples to show top17 significantly up‐regulated mRNAs in activated Lx‐2 cells with siRNA‐DHFR vs. siRNA‐NC. INPP5D up‐regulated in Lx‐2 cells with the decrease of DHFR was tested using QPCR (D) and Western blot (E), **p < 0.01,***p < 0.0001 between indicated groups

In order to clarify the downstream regulators of DHFR in Lx‐2 cells, RNA‐seq was performed in activated Lx‐2 cells with or without DHFR silence and bioinformatics analysis was performed to analyse the differentially expressed RNAs between the two groups. Genes meeting the double criteria of *p* < 0.05 and absolute value of log FC > 1.0 were considered to have significant differences between groups (siRNA‐DHFR + TGF‐β vs. siRNA‐NC + TGF‐β). Based on the results of RNA‐seq and bioinformatics analysis, volcano map and cluster map were drawn respectively (Figure 5A,B). The results demonstrated that there were 27 mRNAs with significant differences among which 18 of which were significantly up‐regulated and 9 were significantly decreased. Then, cluster analysis on the top genes with the most significant up‐regulation and down‐regulation was done respectively again, and the population of top up‐regulated genes was focussed on Figure 5C.

Notably, INPP5D (also known as SHIP1, a potent negative regulator of the PI3K pathway which correlates inversely with histological stages of liver fibrosis) was up‐regulated significantly (Log_2_ fold change = 1.08, *p* = 0.0128) in activated Lx‐2 cells with DHFR silence when compared with that in activated Lx‐2 cells without DHFR silence (siRNA‐DHFR + TGF‐β vs. siRNA‐NC + TGF‐β). Subsequently, QPCR and Western blot were used to verify the results in cell models. The results showed that when DHFR was knocked‐down in Lx‐2 cells, the relative expression of INPP5D was up‐regulated significantly which was consistent with the RNA‐seq result (Figure 5C,D).

### 3.6 INPP5D silence restored the effect remission induced by DHFR knock‐down

**FIGURE 6:**
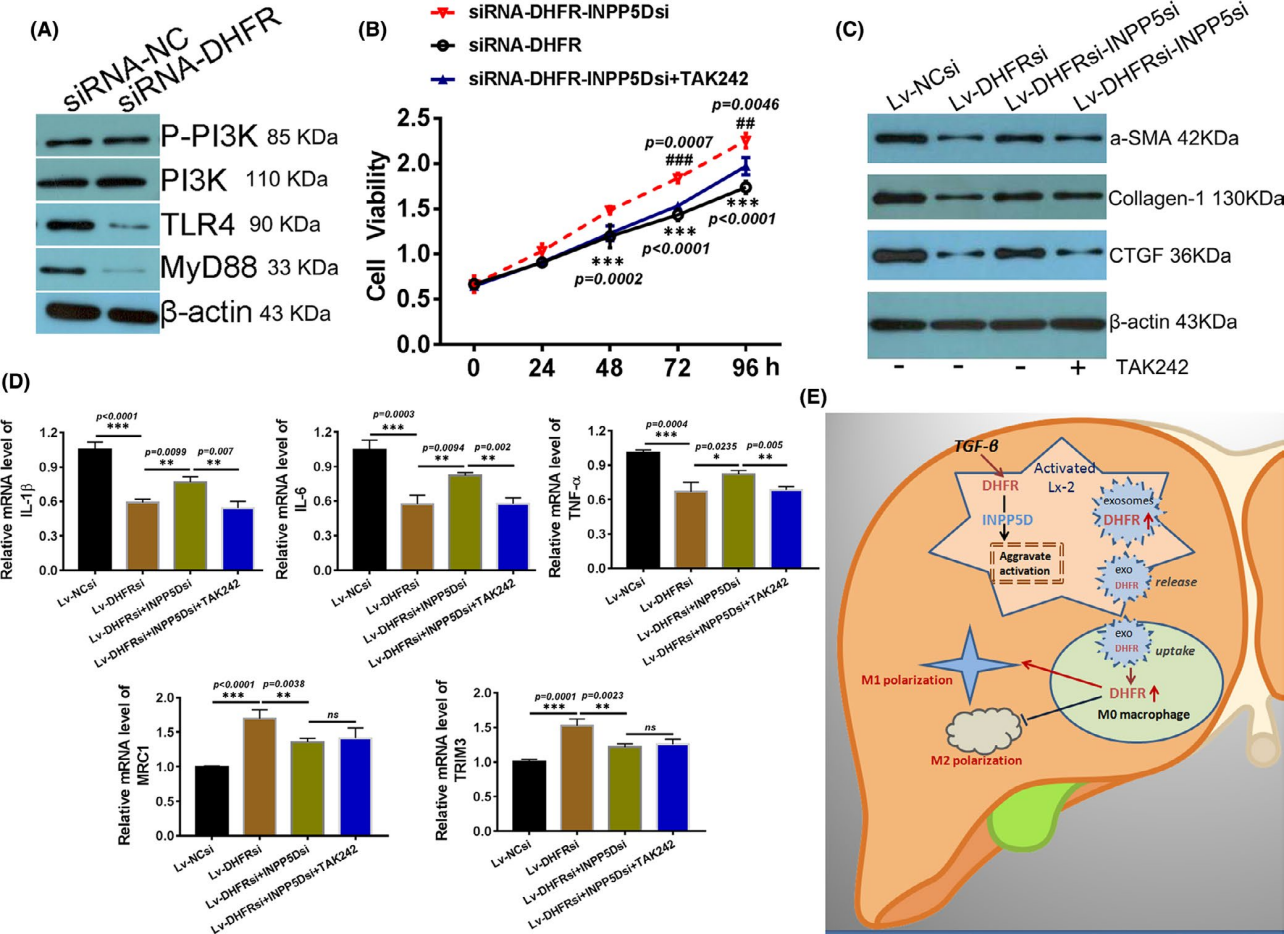
INPP5D silence restored the effect remission induced by DHFR knock‐down. (A) Protein expression of Lx‐2 activation markers was tested in DHFR‐silenced Lx‐2 cells with (Lv‐DHFRsi‐INPP5Dsi) without INPP5D silence (Lv‐DHFRsi). (B) Cell viability of DHFR loss‐of‐function Lx‐2 cells with or without INPP5D silence. As to TLR4 inhibition group, 20 μM TAK242 was used to pretreat the cells for 4 h. * There was significant difference between siRNA‐DHFR and groups; ^#^There was significant difference between Lv‐DHFRsi‐ ‐INPP5Dsi and Lv‐DHFRsi‐INPP5Dsi + TAK242 groups; (C) Expression of Lx‐2 activation markers was tested using Western blot in DHFR‐silenced Lx‐2 cells with (Lv‐DHFRsi‐INPP5Dsi) without INPP5D silence (Lv‐DHFRsi). (D) Relative mRNA levels of M1 and M2 macrophage markers were tested using QPCR in M0 macrophages absorbed exosomes from DHFR‐silenced Lx‐2 with (Lv‐DHFRsi‐INPP5Dsi) or without (Lv‐DHFRsi) INPP5D knock‐down. *, there was significant difference, ns, there was no significant difference between indicated groups. (E) Schematic summary of the anti‐fibrotic effect of DHFR in hepatic stellate cells and the crosstalk with M0 macrophages

It has been confirmed INPP5D was one of the downstream regulators of DHFR in Lx‐2 cells from molecular and protein level. It was reported that INPP5D is a TLR2/TLR4 inhibitor which restrains LPS (TLR4‐ligand)‐induced proinflammatory cytokine production.^27^ Based on these, the key signalling pathway factors were detected in LX‐2 cells with DHFR knock‐down. The Western blot assay demonstrated there was no significant difference in the expression of p‐PI3K, but TLR4 and its adaptor protein MyD88 were significantly reduced in LX‐2 cells with DHFR knock‐down (Lv‐DHFRsi) when compared with control group (Lv‐NCsi) (Figure 6A). These results demonstrated that DHFR may target INPP5D and exert its biological functions through TLR4/MyD88 pathway. Next, INPP5D was silenced in LX‐2 cells with DHFR knock‐down to investigate the changes in cell proliferation and activation capacity. At the same time, TLR4 inhibitor group (Lv‐DHFRsi‐INPP5Dsi + TAK242) was also established.^28^ The CCk‐8 result demonstrated that when INPP5D was silenced in LX‐2 cells with DHFR knock‐down (Lv‐DHFRsi‐INPP5D siRNA), the proliferation ability of Lx‐2 was significantly restored when compared with DHFR knock‐down cells without INPP5D silence (Lv‐DHFRsi). After pretreatment with TAK242, the proliferation promotion caused by INPP5D silence was significantly alleviated again (Figure 6B). In addition, the activation potential was also examined. Being induced by TGF‐β, activation markers were detected by Western blot. The results showed that the expression of α‐SMA, collagen‐I and CTGF was all reduced significantly in TGF‐β‐treated LX‐2 cells with DHFR knock‐down (Lv‐DHFRsi) when compared with control group (Lv‐NCsi), which were all increased obviously when INPP5D was silenced in LX‐2 cells with DHFR knock‐down (Lv‐DHFRsi‐INPP5D siRNA). However, when TLR4 was inhibited, the expression of activation markers decreased significantly again when compared with Lv‐DHFRsi‐INPP5D siRNA group (Figure 6C). These results suggested that DHFR/INPP5D/TLR4 is one of the functional axes of DHFR in regulating Lx‐2 activation.

INPP5D has been reported to affect the release of inflammatory cytokines of macrophages.^29^ To evaluate the effects of INPP5D on M0 macrophage transpolarization, relative mRNA levels of M1 and M2 macrophage markers were detected using qPCR in M0 macrophages. The results showed that M1 markers (IL‐1β, IL‐6 and TNF‐α) were all reduced significantly in M0 macrophage‐absorbed exosomes from Lx‐2 with DHFR knock‐down (Lv‐DHFRsi) when compared with NC group (Lv‐NCsi), but were increased obviously when INPP5D was silenced in Lx‐2 cells with DHFR knock‐down (Lv‐DHFRsi‐INPP5Dsi). At the same time, M2 markers (MRC1 and TRIM3) showed opposite changes. When TAK242 was added during M0 macrophage polarization, the relative mRNA levels of M1 markers dropped obviously again, but there were no significant changes in M2 markers (Figure 6D). These results suggested that INPP5D is also a key downstream target in the polarization of M0 macrophages induced by DHFR and DHFR/INPP5D/TLR4 is one of the functional axes of DHFR in regulating M0‐M1 transpolarization of macrophage. Finally, a comprehensive picture of the molecular mechanisms elucidated in this study was drawn in Figure 6E.

## 4 DISCUSSION

Hepatic fibrosis is accompanied by the accumulation of increased numbers of myofibroblasts in the liver, and the major source of myofibroblast is HCSs. Hepatic macrophages produce cytokines and chemokines that directly influence behaviour of HSCs such as HSCs activation, promotion of aHSCs‐derived myofibroblast proliferation and survival.

Exosomes are critical in cell‐to‐cell communication during the progression of liver disease, and some exosomes were reported to participate in the pathogenesis of liver fibrosis by modulating HSC activation^30^ But the mechanism of exosomes derived from aHSCs involved in liver fibrosis is poorly understood.^31^ Our data showed that exosomes from aHSCs influence polarization of M0 macrophage through delivering exosomal DHFR which would lead to M1 polarization enhancement. However, M1 macrophages secreted proinflammatory cytokines and chemokines to activate and aggravate inflammatory response in liver injury.^32^ Our research indicated the interaction between aHSCs and M1 macrophage mediated through exosomes in the development of liver fibrosis. To explore the key factors in the exosomes derived from activated Lx‐2 which could participate in M1 macrophage polarization, exo‐mRNA‐seq was performed and DHFR came to our eyes as the most significant differential mRNA between groups which expression may be related to the progression of liver cancer through related information disclosed in TCGA. Hence, we supposed that DHFR exerts important function in liver fibrosis. However, few studies focus on the functional role of DHFR in liver fibrosis until now.

A study on methotrexate (MTX)‐induced pulmonary fibrosis found that DHFR knock‐down significantly attenuated MTX‐induced epithelial‐mesenchymal transition (EMT) in A549 cells.^33^ Another study found that chalcone compounds exerted anti‐proliferation and anti‐inflammatory effects on cells through double‐targeting DHFR and thioredoxin reductase (TrxR).^34^ These results indicated DHFR plays an important role in the fibrosis process which was closely related to EMT and inflammation. In this study, DHFR was found significantly up‐regulated in exosomes from activated Lx‐2 cells suggested that DHFR could be positively related to liver fibrosis. DHFR silence led to decrease in cell viability of activated Lx‐2 cells and M1 macrophage polarization, as well as liver fibrosis remission in CCL‐4‐induced liver fibrosis mice. These results are also consistent with the previous research conclusions to a certain extent.

To clarify the downstream genes targeting which DHFR exerts regulation on liver fibrosis, the mRNA‐seq was performed and from which INPP5D was identified as a typical negative target genes of DHFR in activated Lx‐2 cells. INPP5D (also known as SHIP) could restrain proinflammatory cytokine production and correlate inversely with histological stages of liver fibrosis in vivo.^27^, ^35^ Besides, previous researches indicated that INPP5D‐deficient mice were prone to spontaneous inflammation and fibrosis in the lung and intestine, and participated in regulating macrophage polarization.^36^, ^37^ Our rescue cell model by further knock‐down of INPP5D in DHFR‐silenced Lx‐2 was established. The cell function experiments of the rescue model confirmed that INPP5D knock‐down restored the effect remission induced by DHFR silence in both Lx‐2 activation and exosomal‐mediated M1 polarization in M0 macrophages. These results were consistent with previous study that INPP5D expression had a significant inverse correlation with liver fibrosis.^27^

Considering the result that DHFR negatively regulated INPP5D in liver fibrogenesis, the potential mechanism of DHFR regulating INPP5D expression was not fully explored in this study. Although we tried to explore this potential mechanism both through bioinformatics analysis or literature retrieval, unfortunately, DHFR is neither a transcriptional regulator nor an epigenetic regulator because of which there is little relevant information. Only the following information is available for reference: DHFR silence resulted in inhibition of folate metabolism then led to enhancement of activators of PI3K signalling.^38^ Meanwhile, INPP5D was found to be one of the subsequent co‐activators after PI3K activation.^39^ In addition, the regulatory mechanism between DHFR and INPP5D would be involved more complex network, similar to competitive RNA and so on, which needs further scientific exploration.

The data from TCGA database about the DHFR in liver primary tumour samples and its expression on liver cancer patients' survival were analysed. The results indicated DHFR was obviously up‐regulated in tumour group and its expression was negatively correlated with survival probability of liver cancer patients. DHFR expression was tested in liver fibrosis tissues, liver cirrhosis and liver cancer respectively for the first time. It was found DHFR was gradually enhanced from liver fibrosis, liver cirrhosis to liver cancer in terms of the overall trend. These results provided a new understanding for DHFR in the development from liver fibrosis to liver cancer, maybe DHFR will become a promising therapeutic target in liver fibrosis, as well as a predicted and prognosis biomarker of liver cancer.

## CONFLICT OF INTEREST

The authors declare that they have no competing interests.

## AUTHOR CONTRIBUTIONS

**Yu Peng:** Data curation (equal); Formal analysis (equal); Investigation (equal); Software (equal); Supervision (equal); Validation (equal); Visualization (equal). **Zedong Li:** Data curation (supporting); Formal analysis (equal); Software (supporting); Visualization (supporting). **Sheng Chen:** Validation (supporting); Visualization (supporting). **Jun Zhou:** Conceptualization (lead); Funding acquisition (lead); Methodology (lead); Project administration (lead); Resources (lead); Writing‐original draft (lead); Writing‐review & editing (lead).

## Supporting information

Supplementary Material

## Data Availability

The data sets used and/or analysed during the current study are available from the corresponding author on reasonable request.

## References

[1] Aydin MM , Akcali KC . Liver fibrosis. Turk J Gastroenterol. 2018;29(1):14‐21. 29391303 10.5152/tjg.2018.17330 PMC6322608

[2] Altamirano‐Barrera A , Barranco‐Fragoso B , Mendez‐Sanchez N . Management strategies for liver fibrosis. Ann Hepatol. 2017;16(1):48‐56. 28051792 10.5604/16652681.1226814

[3] Wandzioch E , Kolterud A , Jacobsson M , Friedman SL , Carlsson L . Lhx2‐/‐ mice develop liver fibrosis. Proc Natl Acad Sci USA. 2004;101(47):16549‐16554. 15536133 10.1073/pnas.0404678101 PMC526277

[4] Senoo H , Mezaki Y , Fujiwara M . The stellate cell system (vitamin A‐storing cell system). Anat Sci Int. 2017;92(4):387‐455. 28299597 10.1007/s12565-017-0395-9

[5] Koyama Y , Brenner DA . Liver inflammation and fibrosis. J Clin Investig. 2017;127(1):55‐64. 28045404 10.1172/JCI88881 PMC5199698

[6] Campana L , Iredale JP . Regression of liver fibrosis. Semin Liver Dis. 2017;37(1):1‐10. 28201843 10.1055/s-0036-1597816

[7] Tarique AA , Logan J , Thomas E , Holt PG , Sly PD , Fantino E . Phenotypic, functional, and plasticity features of classical and alternatively activated human macrophages. Am J Respir Cell Mol Biol. 2015;53(5):676‐688. 25870903 10.1165/rcmb.2015-0012OC

[8] Shapouri‐Moghaddam A , Mohammadian S , Vazini H , et al. Macrophage plasticity, polarization, and function in health and disease. J Cell Physiol. 2018;233(9):6425‐6440. 10.1002/jcp.26429 PMC13482560 29319160

[9] Maia J , Caja S , Strano Moraes MC , Couto N , Costa‐Silva B . Exosome‐based cell‐cell communication in the tumor microenvironment. Front Cell Dev Biol. 2018;6:18. 29515996 10.3389/fcell.2018.00018 PMC5826063

[10] Schorey JS , Cheng Y , Singh PP , Smith VL . Exosomes and other extracellular vesicles in host‐pathogen interactions. EMBO Rep. 2015;16(1):24‐43. 25488940 10.15252/embr.201439363 PMC4304727

[11] Bang C , Thum T . Exosomes: new players in cell‐cell communication. Int J Biochem Cell Biol. 2012;44(11):2060‐2064. 22903023 10.1016/j.biocel.2012.08.007

[12] Guo P , Yu H , Wang Y , Xie X , Chen G . Exosome: an emerging participant in the development of liver disease. Hepat Mon. 2017;17(8):e58021.

[13] Sung S , Kim J , Jung Y . Liver‐derived exosomes and their implications in liver pathobiology. Int J Mol Sci. 2018;19(12):3715. 10.3390/ijms19123715 PMC6320937 30469540

[14] Wang L , Wang Y , Quan J . Exosomes derived from natural killer cells inhibit hepatic stellate cell activation and liver fibrosis. Hum Cell. 2020;33(3):582‐589. 32449114 10.1007/s13577-020-00371-5

[15] Wang L , Wang Y , Quan J . Exosomal miR‐223 derived from natural killer cells inhibits hepatic stellate cell activation by suppressing autophagy. Mol Med. 2020;26(1):81. 32873229 10.1186/s10020-020-00207-w PMC7465359

[16] Chen L , Brenner DA , Kisseleva T . Combatting fibrosis: exosome‐based therapies in the regression of liver fibrosis. Hepatol Commun. 2019;3(2):180‐192. 30766956 10.1002/hep4.1290 PMC6357832

[17] Baig MS , Roy A , Rajpoot S , et al. Tumor‐derived exosomes in the regulation of macrophage polarization. Inflamm Res. 2020;69(5):435‐451. 32162012 10.1007/s00011-020-01318-0

[18] Pritchard A , Tousif S , Wang Y , et al. Lung tumor cell‐derived exosomes promote M2 macrophage polarization. Cells. 2020;9(5):1303. 10.3390/cells9051303 PMC7290460 32456301

[19] He X , Dong Z , Cao Y , et al. MSC‐derived exosome promotes m2 polarization and enhances cutaneous wound healing. Stem Cells Int. 2019;2019:7132708. 31582986 10.1155/2019/7132708 PMC6754952

[20] Xu R , Zhang F , Chai R , et al. Exosomes derived from pro‐inflammatory bone marrow‐derived mesenchymal stem cells reduce inflammation and myocardial injury via mediating macrophage polarization. J Cell Mol Med. 2019;23(11):7617‐7631. 31557396 10.1111/jcmm.14635 PMC6815833

[21] Heo JS , Choi Y , Kim HO . Adipose‐derived mesenchymal stem cells promote M2 macrophage phenotype through exosomes. Stem Cells Int. 2019;2019:7921760. 31781246 10.1155/2019/7921760 PMC6875419

[22] Song M , Han L , Chen FF , et al. Adipocyte‐derived exosomes carrying sonic Hedgehog mediate M1 macrophage polarization‐induced insulin resistance via Ptch and PI3K pathways. Cell Physiol Biochem. 2018;48(4):1416‐1432. 30064125 10.1159/000492252

[23] Liu H , Qin Y , Zhai D , et al. Antimalarial drug pyrimethamine plays a dual role in antitumor proliferation and metastasis through targeting DHFR and TP. Mol Cancer Ther. 2019;18(3):541‐555. 30642883 10.1158/1535-7163.MCT-18-0936

[24] Zhou J , Duan L , Huang J , et al. Portable detection of colorectal cancer SW620cells by using a personal glucose meter. Anal Biochem. 2019;577:110‐116. 31034799 10.1016/j.ab.2019.04.018

[25] Chen H , Liu Y , Jiang CJ , Chen YM , Li H , Liu QA . Calcium‐activated chloride channel A4 (CLCA4) plays inhibitory roles in invasion and migration through suppressing epithelial‐mesenchymal transition via PI3K/AKT signaling in colorectal cancer. Med Sci Monit. 2019;25:4176‐4185. 31164625 10.12659/MSM.914195 PMC6563650

[26] Zhou J , Qu G , Zhang G , et al. Glycerol kinase 5 confers gefitinib resistance through SREBP1/SCD1 signaling pathway. J Exp Clin Cancer Res. 2019;38(1):96. 30791926 10.1186/s13046-019-1057-7 PMC6385389

[27] Katsounas A , Trippler M , Kottilil S , Lempicki RA , Gerken G , Schlaak JF . Altered expression of SHIP, a Toll‐like receptor pathway inhibitor, is associated with the severity of liver fibrosis in chronic hepatitis C virus infection. J Infect Dis. 2011;204(8):1181‐1185. 21917890 10.1093/infdis/jir500 PMC3173502

[28] Long T , Liu Z , Shang J , et al. Polygonatum sibiricum polysaccharides play anti‐cancer effect through TLR4‐MAPK/NF‐kappaB signaling pathways. Int J Biol Macromol. 2018;111:813‐821. 29343453 10.1016/j.ijbiomac.2018.01.070

[29] Yang L , Liu L , Ying H , et al. Acute downregulation of miR‐155 leads to a reduced collagen synthesis through attenuating macrophages inflammatory factor secretion by targeting SHIP1. J Mol Histol. 2018;49(2):165‐174. 29330743 10.1007/s10735-018-9756-5

[30] Zhang XW , Zhou JC , Peng D , et al. Disrupting the TRIB3‐SQSTM1 interaction reduces liver fibrosis by restoring autophagy and suppressing exosome‐mediated HSC activation. Autophagy. 2020;16(5):782‐796. 31286822 10.1080/15548627.2019.1635383 PMC7144866

[31] Wan L , Xia T , Du Y , et al. Exosomes from activated hepatic stellate cells contain GLUT1 and PKM2: a role for exosomes in metabolic switch of liver nonparenchymal cells. FASEB J. 2019;33(7):8530‐8542. 30970216 10.1096/fj.201802675R

[32] Sun YY , Li XF , Meng XM , Huang C , Zhang L , Li J . Macrophage phenotype in liver injury and repair. Scand J Immunol. 2017;85(3):166‐174. 27491503 10.1111/sji.12468

[33] Kawami M , Honda N , Hara T , Yumoto R , Takano M . Investigation on inhibitory effect of folic acid on methotrexate‐induced epithelial‐mesenchymal transition focusing on dihydrofolate reductase. Drug Metab Pharmacokinet. 2019;34(6):396‐399. 31601464 10.1016/j.dmpk.2019.08.003

[34] Gan FF , Zhang R , Ng HL , et al. Novel dual‐targeting anti‐proliferative dihydrotriazine‐chalcone derivatives display suppression of cancer cell invasion and inflammation by inhibiting the NF‐kappaB signaling pathway. Food Chem Toxicol. 2018;116(Pt B):238‐248. 29630947 10.1016/j.fct.2018.04.003

[35] Dobranowski P , Sly LM . SHIP negatively regulates type II immune responses in mast cells and macrophages. J Leukoc Biol. 2018;103:1053‐1064. 10.1002/JLB.3MIR0817-340R 29345374

[36] Rauh MJ , Ho V , Pereira C , et al. SHIP represses the generation of alternatively activated macrophages. Immunity. 2005;23(4):361‐374. 16226502 10.1016/j.immuni.2005.09.003

[37] Qin S , Li J , Zhou C , et al. SHIP‐1 regulates phagocytosis and M2 polarization through the PI3K/Akt‐STAT5‐Trib1 circuit in pseudomonas aeruginosa infection. Front Immunol. 2020;11:307. 32256487 10.3389/fimmu.2020.00307 PMC7093384

[38] Klinghoffer RA , Frazier J , Annis J , et al. A lentivirus‐mediated genetic screen identifies dihydrofolate reductase (DHFR) as a modulator of beta‐catenin/GSK3 signaling. PLoS One. 2009;4(9):e6892. 19727391 10.1371/journal.pone.0006892 PMC2731218

[39] Cojohari O , Mahmud J , Altman AM , Peppenelli MA , Miller MJ , Chan GC . Human cytomegalovirus mediates unique monocyte‐to‐macrophage differentiation through the PI3K/SHIP1/Akt signaling network. Viruses. 2020;12(6):652. 10.3390/v12060652 PMC7354488 32560319

