# Supplementary material for: DHFR silence alleviated the development of liver fibrosis by affecting the crosstalk between hepatic stellate cells and macrophages
Source: J Cell Mol Med. 2021 Oct 9;25(21):10049–60. doi: 10.1111/jcmm.16935 (PMC8572769; doi:10.1111/jcmm.16935)
Supplement: Supplementary file 1 — Supplementary Material [file JCMM-25-10049-s001.pdf]

Relative gray value (vs β -actin) was shown in red

Fig 1C

	Con	TGF-β		
α-SMA	96663	173652	90189	159028
	83715	144404		
CTGF	61720	116832	59476	114050
	57232	111268		
Collagen-I	61388	103953	61965.5	103767
	62543	103581		
β-actin	256343	222183	256817.5	219188.5
	257292	216194		
	Con	TGF-β		
α-SMA	0.351179339	0.725530765		
CTGF	0.231588579	0.520328393		
Collagen-I	0.241282233	0.473414436		

Fig 1D

	Cl-con	CL-TGF-β	Ex-con	EX-TGF-β
CD81	39425	37278	100944	160753
	48216	54976	136580	176030
TSG101	40301	47606	72266	55709
	23261	42607	72612	70835
Calnexin	49032	65235	0	0
	39398	61619	0	0

Fig IH

	Ex-NC	EX-TGF-β	Ex-con	EX-TGF-β
IL-1β	81856	127426	0.820414136	1.78283012
IL-6	268875	322278	2.694840339	4.509024261
CCR7	82817	124480	0.830045904	1.741612335
TNF-α	180130	273861	1.805380159	3.831617092
β-actin	99774	71474		

Fig 1I

	Ex-NC	EX-TGF-β	Ex-con	EX-TGF-β
MRC1	138687	108452	1.286807824	0.930327517
TIMP3	162214	111641	1.505103177	0.957683531
β-actin	107776	116574		

Fig 3E

	siRNA-NC	siRNA-NC+TGF	siRNA-DHFR	siRNA-DHFR+TGF
DHFR	187122	223590	117603	125858
α-SMA	202223	354925	270565	261269
CTGF	91540	128676	142605	79917
Collagen-I	119053	134530	112059	96557
β-actin	158462	150255	150614	157977
	siRNA-NC	siRNA-NC+TGF	siRNA-DHFR	siRNA-DHFR+TGF
DHFR	1.180863551	1.488070281	0.780823828	0.796685593
α-SMA	1.276160846	2.36215101	1.796413348	1.653842015
CTGF	0.577677929	0.856384147	0.946824332	0.505877438
Collagen-I	0.751303152	0.895344581	0.744014501	0.611209227

Fig 3F

**Exosome**	siRNA-NC	siRNA-NC+TGF	siRNA-DHFR	siRNA-DHFR+TGF
IL-1β	147602	216472	150727	165243
IL-6	292983	363906	299366	281052
CCR7	500857	615946	586744	554365
TNF-α	178746	243724	204850	176992
β-actin	281080	284338	267519	281915
	siRNA-NC	siRNA-NC+TGF	siRNA-DHFR	siRNA-DHFR+TGF
IL-1β	0.52512452	0.761319275	0.563425402	0.58614476
IL-6	1.042347374	1.27983597	1.11904575	0.996938794
CCR7	1.78190195	2.166245806	2.19327973	1.96642605
TNF-α	0.635925715	0.857162954	0.765740004	0.627820442

Fig 4D

	DMSO	Lv-siRNA-NC	Lv-siRNA-DHFR

Clo3α 1	44527	94672	70797
Clo4α 1	51284	80441	62318
TIMP1	65140	119366	78810
β-actin	85122	85454	84623
	DMSO	Lv-siRNA-NC	Lv-siRNA-DHFR
Clo3α 1	0.523096262	1.107870901	0.836616523
Clo4α 1	0.602476446	0.94133686	0.736419177
TIMP1	0.765254576	1.396845086	0.931307091

Fig 5E

	siRNA-NC	siRNA-DHFR	siRNA-NC	siRNA-DHFR
DHFR	426740	250358	1.078527759	0.71884529
INPP5D	406640	516395	1.027727722	1.482709215
β-actin	395669	348278		

Fig 6C

	Lv-NCsi	Lv-DHFRsi	Lv-DHFRsi-INPP5Dsi	Lv-DHFRsi-INPP5Dsi+TAK242
α-SMA	1010667	626561	853105	602750
CTGF	864209	525059	753961	641194
Collagen-I	1512456	897600	1321369	788612
β-actin	929569	871534	885423	889469
	Lv-NCsi	Lv-DHFRsi	Lv-DHFRsi-INPP5Dsi	Lv-DHFRsi-INPP5Dsi+TAK242
α-SMA	1.087242582	0.718917449	0.963499932	0.677651498
CTGF	0.929687845	0.602453834	0.851526333	0.72087279
Collagen-I	1.627050816	1.029908185	1.492359019	0.886609876

Fig 6A

	siRNA-NC	siRNA-DHFR	siRNA-NC	siRNA-DHFR	
p-PI3K	109183	110904	1.001338995	1.038329744	vs PI3K
PI3K	109037	106810	0.544700217	0.503497756	
TLR4	109683	75341	0.547927345	0.355154241	
MyD88	96359	44937	0.481366584	0.211831089	
β-actin	200178	212136			
